# Targeted Hsp70 expression combined with CIK-activated immune reconstruction synergistically exerts antitumor efficacy in patient-derived hepatocellular carcinoma xenograft mouse models

**DOI:** 10.18632/oncotarget.2835

**Published:** 2014-11-26

**Authors:** Huanzhang Hu, Yinghe Qiu, Minggao Guo, Yao Huang, Lin Fang, Zhangxiao Peng, Weidan Ji, Yang Xu, Shuwen Shen, Yan Yan, Xuandong Huang, Junnian Zheng, Changqing Su

**Affiliations:** ^1^ Department of Hepatobiliary Surgery, Fuzhou General Hospital of Nanjing Military Area, Fuzhou, China; ^2^ Department of Molecular Oncology & Biliary Tract Surgery, Eastern Hepatobiliary Surgical Hospital & National Center of Liver Cancer, Second Military Medical University, Shanghai, China; ^3^ Department of Surgery, Shanghai Sixth People Hospital, Shanghai Jiao-Tong University, Shanghai, China; ^4^ Jiangsu Center for the Collaboration and Innovation of Cancer Biotherapy, Xuzhou Medical College, Xuzhou, China; ^5^ Department of Oncological Surgery, Second People’s Hospital of Huai’an, Jiangsu Province, China

**Keywords:** Patient-derived tumor xenograft model, Hepatocellular carcinoma, Onolytic adenovirus, Heat shock protein, Cytokine-induced killer

## Abstract

The patient-derived tumor xenograft (PDTX) models can reproduce a similar natural genetic background and similar biological behaviors to tumor cells in patients, which is conducive to the assessment of personalized cancer treatment. In this study, to verify the targeting and effectiveness of the therapeutic strategy using a Survivin promoter-regulated oncolytic adenovirus expressing Hsp70, the PDTX models of hepatocellular carcinoma (HCC) were established in nude mice and the cytokine-induced killer (CIK) cells were intravenously infused into mice to partially reconstruct the mouse immune function. The results demonstrated that, either the immune anti-tumor effect caused by CIK cell infusion or the oncolytic effect generated by oncolytic adenovirus replication was very limited. However, the synergistic tumor inhibitory effect was significantly enhanced after treatments with oncolytic adenovirus expressing Hsp70 combined with CIK cells. Oncolytic adenovirus mediated the specific expression of Hsp70 in cancer tissues allowed the CIK chemotaxis, and induce the infiltration of CD3+ T cells in tumor stroma, thereby exhibiting anti-tumor activity. The anti-tumor effect was more effective for the highly malignant tumor xenografts with highly Survivin expression. This strategy can synergistically activate multiple anti-tumor mechanisms and exert effective anti-tumor activities that have a significant inhibitory effect against the growth of HCC xenografts.

## INTRODUCTION

Hepatocellular carcinoma (HCC) is a common malignant tumor in China. Conventional surgical treatment, radiation therapy and chemotherapy have shown poor efficacies in treating HCC and result in a high recurrence rate of HCC. The comprehensive therapy combined with various adjuvant therapeutic strategies has often been applied to improve efficacy and prognosis in clinical practice. Among the comprehensive treatment of HCC, gene therapy and cell therapy have become important adjuvant treatments. In our preliminary study, two technical platforms for broad spectral tumor-targeting gene therapy were established. One method used an oncolytic adenovirus regulated by a tumor-specific Survivin gene promoter to mediate the targeting expression of Hsp70 or P53 gene [1,2], the other method involved using an oncolytic adenovirus regulated by the carcinoembryonic antigen (CEA) gene promoter to mediate the targeting expression of Hsp70 gene [3]. These two gene therapies mediated by oncolytic adenoviruses demonstrated good efficacies in the *in vivo* and *in vitro* experiments for various tumors. When the gene targeting therapeutic strategy of Hsp70 gene expression mediated by the Survivin promoter-regulated oncolytic adenovirus was applied to HCC treatment, it was found that the oncolytic adenovirus could obtain a high proliferative activity and a high expression level of Hsp70 in Survivin-positive HCC, and it could strengthen the killing effect on cancer cells without significant toxic exposure for normal cells [4]. This phenomenon occurs because the Survivin promoter can regulate the expression of adenoviral replication gene and, consequently, restrict the replication of adenovirus in tumor cells. The large amount of replicated viruses lyse tumor cells (oncolytic effect), while the progeny virions are released to infect more surrounding tumor cells [5-7]. Moreover, the viral replication increases the copy number of anti-tumor genes it carries, resulting in high expression efficiency and a stronger anti-tumor effect. Therefore, the targeting gene therapeutic strategy of anti-tumor gene expression mediated by oncolytic adenoviruses can achieve a synergistic oncolytic effect caused by viral replication and the anti-tumor effect as a result of the high anti-tumor gene expression, leading to an improvement in the efficacy and safety of the anti-tumor treatment.

Screening for anti-tumor genes is important to improve the efficacy of tumor therapy. Heat or other stimulation by adverse factors may cause a stress response in tumor cells and induce the expression of heat shock proteins (Hsp), in which Hsp70 can induce a specific immune response against tumor cells and serve as a molecular target for natural killer (NK) to recognize cancer cells [8-11]. As a potent anti-tumor factor, Hsp70 can become an effective candidate gene in anti-tumor immunity. However, HCC cells are highly malignant and proliferate extensively, so the oncolytic capability of oncolytic adenoviruses or the anti-tumor effect of the targeted gene alone is inadequate to completely stop the growth of tumors. This limitation requires us to further optimize the gene targeting therapeutic strategy for HCC to enhance the efficacy and safety of the therapeutic system. Since the human-derived tumor cell line xenograft model always is established in immunodeficient nude mice or mice with severe combined immunodeficiency (SCID), and Hsp70 is an immunomodulatory gene, so the immunomodulatory effect of Hsp70 cannot be studied in the immunodeficient xenograft mouse model. In our previous study, cytokine-induced killer (CIK) cells were infused into the immunodeficient mouse model to partially reconstruct the immune function and to investigate the anti-tumor effects of immune regulating genes, which was successful in a gastric cancer xenograft model in nude mice [1]. The establishment of a CIK activated immune reconstitution nude mouse model provided the conditions for our study of human-derived tumor immunomodulatory therapy.

The study of the mechanism of oncogenesis and tumor progression, the research and development of anti-tumor drugs, and the optimization and screening of anti-cancer treatment strategies require animal models that have a similar natural genetic background and similar biological behaviors to tumor cells. In the process of establishing a tumor cell line and its passage, artificial modification of the genetic background may be introduced into, and genetic changes may also occur during the long-term passage, such as karyotype instability including genetic mutations, gene translocation or deletion, which can all lead to some changes in cellular biological behaviors. Therefore, the genetic and biological characteristics of tumor cell line xenografts are very different from the actual clinical tumors, so there may be a selection bias of the anti-tumor drug or the evaluation of a treatment strategy. The patient-derived tumor xenograft (PDTX) model established in recent years not only retains the atypia and structure of primary tumors, but it also displays the similar biological behaviors to primary tumors, which can better reflect the individual tumor characteristics of different patients. This is very significant for individualized tumor research and treatment [12-15]. In this study, the clinical surgical specimens of patients with HCC were used to establish PDTX models. Combined with CIK cell activated immune reconstitution, the treatment simulated a clinical trial in patients and was applied to investigate the efficacy of the targeted Hsp70 expression mediated by oncolytic adenovirus on HCC xenografts.

## RESULTS

### Identification of the cancer-specific replication and gene expression of adenoviruses in HCC cell lines

**Figure 1:**
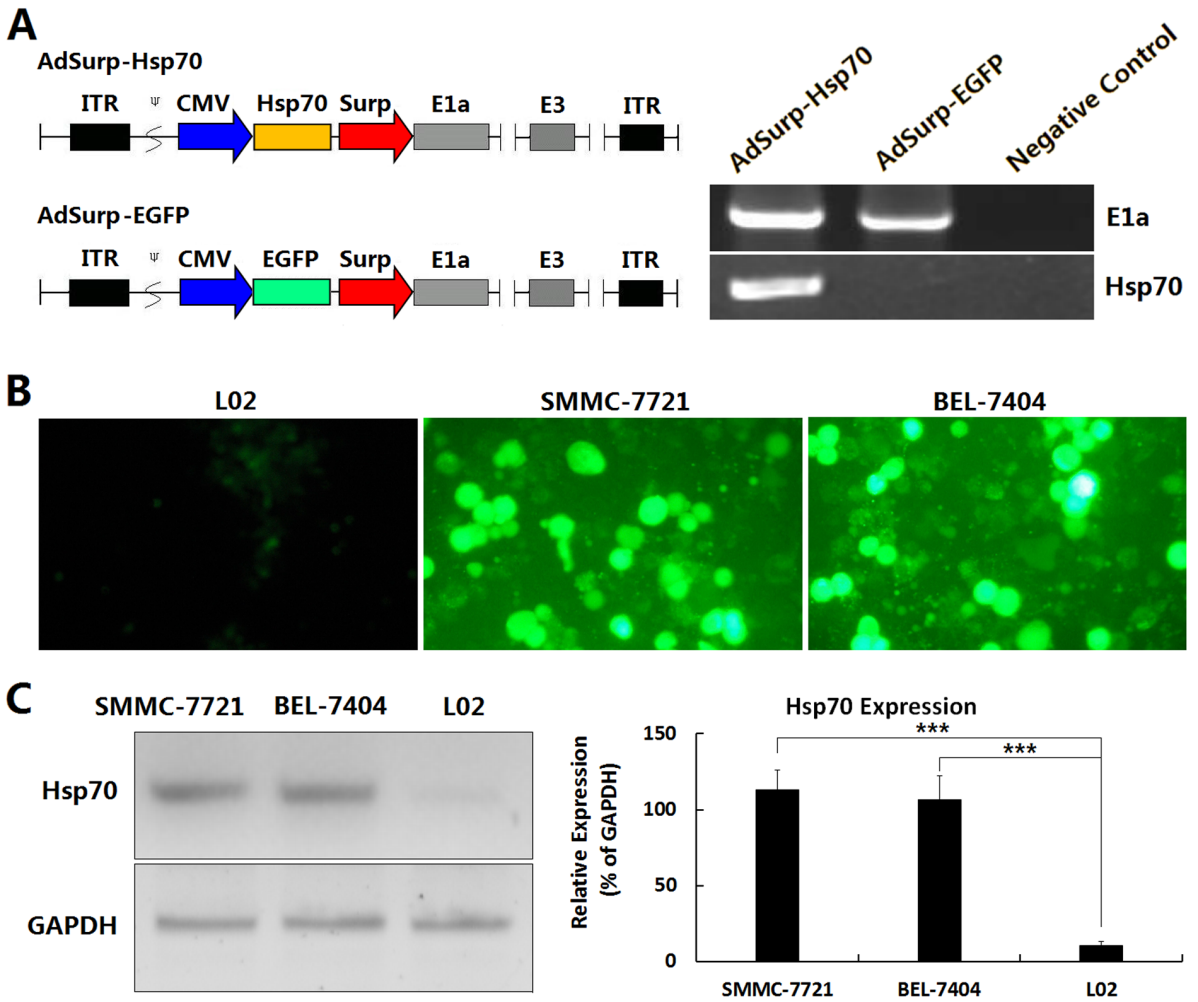
Identification of adenovirus vector in HCC cell lines (A) Schematic diagram of the oncolytic adenoviruses and amplification of the early replication gene E1a as well as the therapeutic gene Hsp70 was performed by PCR in adenoviral vectors AdSurp-Hsp70 and AdSurp-EGFP. The Survivin promoter was used to regulate the adenoviral E1a gene, and the expression cassettes of Hsp70 and EGFP were inserted into the upstream of E1a gene, then generated the recombinant oncolytic adenoviruses AdSurp-Hsp70 and AdSurp-EGFP. ITR: inverted terminal repeats; ψ: adenovirus 5 packaging signal; CMV: cytomegalovirus promoter; Surp: Survivin promoter. (B) The indicated cell lines were seeded into 24-well plates at a concentration of 1×10^5^ cells/well, and infected with AdSurp-EGFP at an MOI of 1 pfu/cell, cultured for 48 h and observed the EGFP-positive cells under a fluorescence microscope, original magnification: ×200. (C) The cell lines were seeded into 24-well plates at a concentration of 1×10^5^ cells/well, and infected with AdSurp-Hsp70 at an MOI of 1 pfu/cell, cultured for 48 h and the expression of Hsp70 was examined by Western blotting; ***P<0.001.

Two pre-constructed Survivin promoter-regulated oncolytic adenoviruses, AdSurp-Hsp70 and AdSurp-EGFP [1,3,4], were amplified in 293 cells and identified by polymerase chain reaction (PCR). The oncolytic adenoviruses AdSurp-Hsp70 and AdSurp-EGFP, regulated by the Survivin promoter, were E1a positive. The adenovirus AdSurp-Hsp70 carrying the Hsp70 gene was Hsp70 positive, while the control virus AdSurp-EGFP was Hsp70 negative (Fig. 1A), suggesting that the structure of the adenoviruses was consistent with expectations.

After infection of adenoviruses, the virus AdSurp-EGFP mediated more EGFP expression in HCC cells than in normal liver cells (Fig. 1B), and the virus AdSurp-Hsp70 mediated more Hsp70 expression in HCC cells than in normal liver cells (Fig. 1C), demonstrating that the adenoviruses have potential to target HCC cells.

### Identification of Survivin expression in HCC specimens

**Figure 2:**
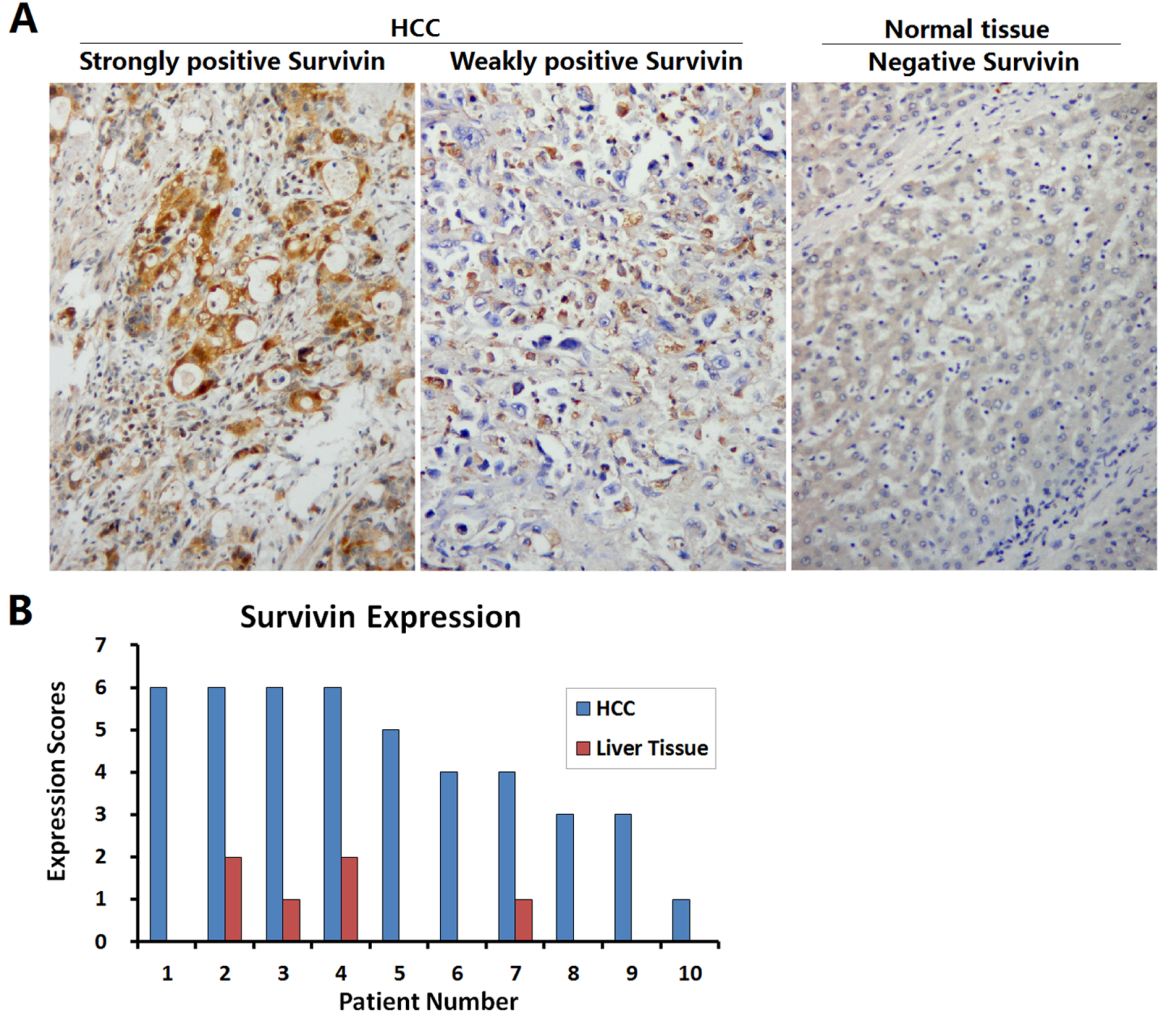
Identification of Survivin expression in HCC specimens (A) HCC specimens and paracancerous liver tissues collected from 10 patients with HCC were fixed in 10% formalin for 6 h to prepare the paraffin-embedded sections, and the expression of Survivin was detected by streptavidin-peroxidase (SP) immunohistochemistry; original magnification: ×200. (B) For each slice, the number of Survivin positive cells was counted within 5 medium-power magnification fields of view (20× objective lens) under microscope. The results were determined by scoring the stained cell ratio and the staining intensity, from 0 to 6 scores.

The HCC specimens and the paracancerous liver tissues were collected from 10 patients undergoing clinical operation, and Survivin expression was detected by immunohistochemistry (Fig. 2A). The expression of Survivin was positive in all 10 HCC specimens, among them 3 were weakly positive and 7 were strongly positive. Whereas, Survivin expression in the corresponding paracancerous liver tissues was significantly weakened, with negative expression in 6 cases and weakly positive expression in 4 cases (Fig. 2B).

### Anti-tumor effect of oncolytic adenovirus-mediated Hsp70 expression on HCC xenograft mouse models

**Figure 3:**
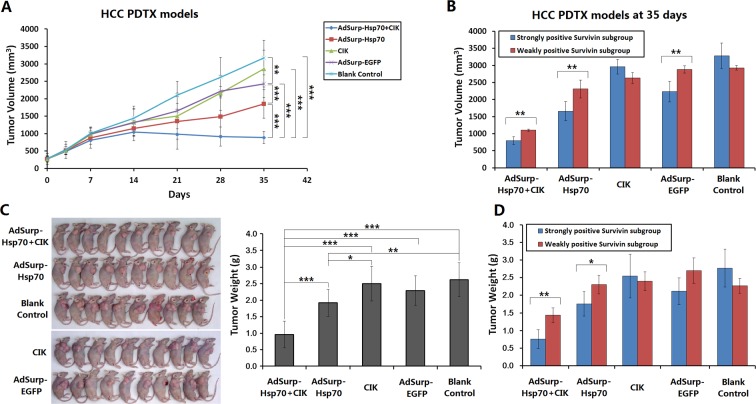
Anti-tumor effect of Hsp70 expression mediated by oncolytic adenovirus in HCC xenograft nude mouse models (A) Fresh HCC tissues from 10 cases of clinical surgical specimens were cut to a depth of 2 mm in diameter and subcutaneously buried in the right axilla of eighty nude mice by a trocar puncture. Mice were assigned to 5 groups (AdSurp-Hsp70+CIK, AdSurp-Hsp70, CIK, AdSurp-EGFP, and the blank control group). After tumor xenografts were formed, mice in the AdSurp-Hsp70+CIK and CIK groups were infused with CIK cells through tail vein to a concentration of 10^7^ cells/mouse. Subsequently, the corresponding viral treatment was given based on the grouping at a total of 1×10^9^ pfu of adenoviruses. The blank control group was injected with the viral preservation solution instead of virus injection. Tumor size was measured regularly, and tumor volume was calculated to result in the tumor growth curves; **P<0.01, ***P<0.001. (B) Each experimental group was divided into a weakly positive Survivin subgroup and a strongly positive Survivin subgroup for further comparison of tumor volume. **P<0.01. (C) After 35 days of the first treatment, the observation was terminated. The tumors were collected and weighed; *P<0.05, **P<0.01, ***P<0.001. (D) Each experimental group was divided into a weakly positive Survivin subgroup and a strongly positive Survivin subgroup for further comparison of tumor weight; *P<0.05, **P<0.01.

Through establishing a PDTX model using 10 cases of HCC surgical specimens, combined with the infusion of CIK cells to partially reconstruct the immune function in nude mice, the killing effect of oncolytic adenovirus-mediated Hsp70 expression on tumor xenografts was observed. After 7 days of viral therapy on the tumor xenografts with a total viral dose of 1×10^9^ pfu, compared with the blank control group, the AdSurp-Hsp70+CIK treatment group showed a significant efficacy. After 14 days, the AdSurp-Hsp70 treatment group showed a certain effect. After 21 days, the CIK and AdSurp-EGFP groups also displayed a certain effect, but the efficacy of CIK treatment gradually disappeared thereafter. Comparison of the anti-tumor efficacy of different groups showed that the efficacy of the AdSurp-Hsp70+CIK treatment group was best, followed by the AdSurp-Hsp70 group. Additionally, the CIK group or AdSurp-EGFP group also exhibited some anti-tumor effect in the later stage (Fig. 3A). Each experimental group was divided into a weakly positive Survivin subgroup and a strongly positive Survivin subgroup for further comparisons, considering the blank control group, the growth of tumor xenografts from the strongly positive Survivin subgroup was slightly faster than that from the weakly positive Survivin subgroup, but this difference was not statistically significant. In the AdSurp-Hsp70+CIK, AdSurp-Hsp70 and AdSurp-EGFP treatment groups, the treatment efficacies for the tumor xenografts in the strongly positive Survivin subgroups were significantly better than those in the weakly positive Survivin subgroups, and the efficacy of the CIK group was not related to Survivin expression (Fig. 3B).

The tumor xenograft model was observed for 35 days since the first treatment. Just then, the tumors in the control group exceeded the criteria (3000 mm^3^) defined by the experimental animal ethics committee, the observation was terminated. The tumors were collected and weighed. The analyses found the smallest average tumor weight was present in the AdSurp-Hsp70+CIK treatment group, followed by the AdSurp-Hsp70 group, these both were significantly different from the control group (Fig.3C). In the AdSurp-Hsp70+CIK, AdSurp-Hsp70, and AdSurp-EGFP treatment groups, the weights of the tumor xenografts for the cases in the strongly positive Survivin subgroup were significantly lower than those in the weakly positive Survivin subgroup (Fig. 3D).

### Targeting replication of oncolytic adenoviruses and specific expression of Hsp70

**Figure 4:**
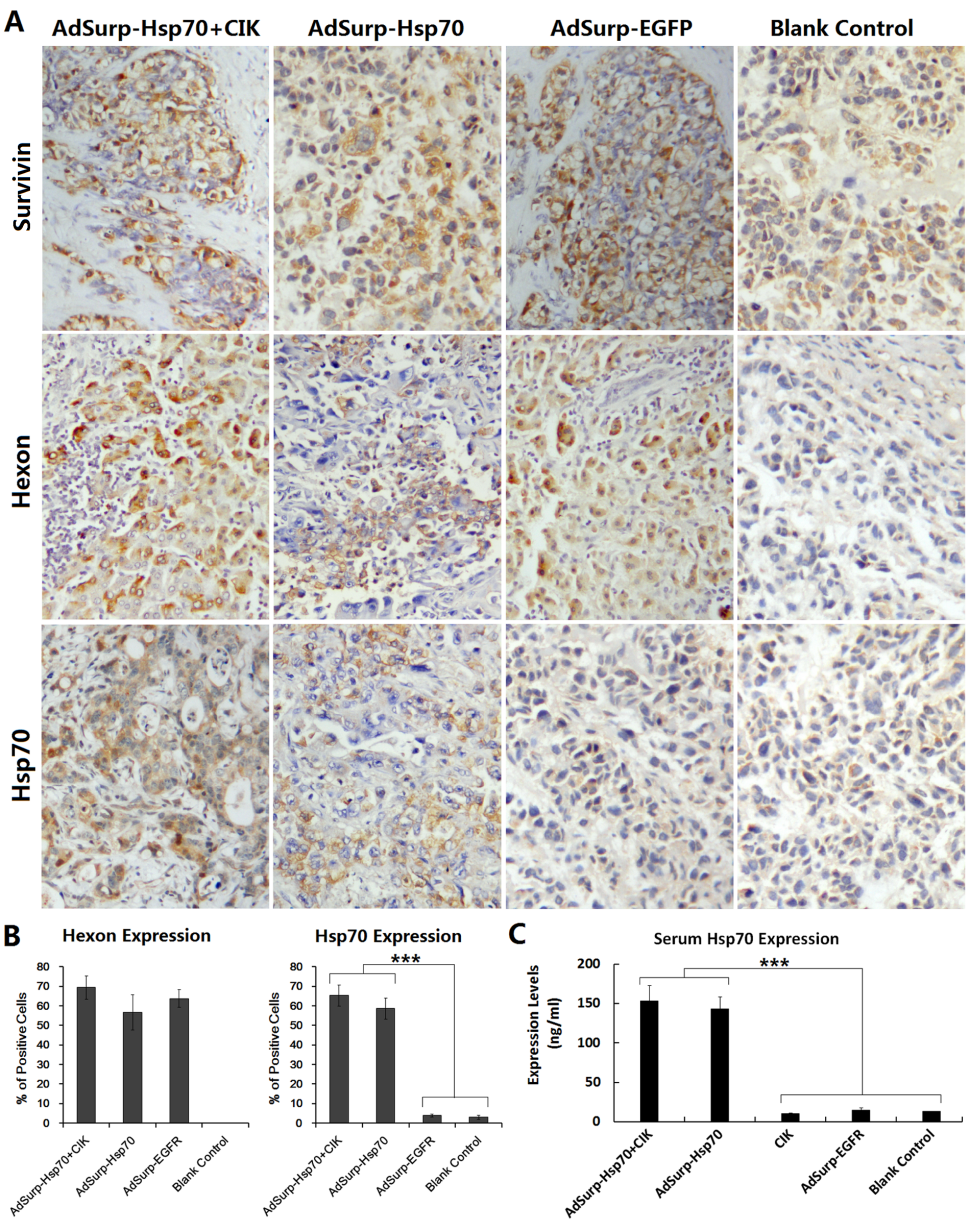
Expression of E1a and Hsp70 mediated by oncolytic adenoviruses (A) Tumor xenografts were fixed in 10% neutralized formalin for 6 h to prepare the paraffin-embedded sections. SP immunohistochemical staining was performed to locate the expressions of indicated proteins; original magnification: ×200. (B) For each slice, the percentages of positive cells were counted within 5 medium-power magnification fields of view (20× objective lens) under microscope. The results were determined by scoring the stained cell ratio and the staining intensity; ***P<0.001. (C) Mouse blood was collected to prepare sera. The expression of Hsp70 protein was detected using an ELISA; ***P<0.001.

Immunohistochemical staining was performed to observe the localization of the expressions of the adenovirus capsid protein Hexon and the therapeutic gene Hsp70. The results showed that, after treatment with oncolytic adenovirus, the expressions of Hexon and Hsp70 in the AdSurp-Hsp70+CIK and AdSurp-Hsp70 groups, as well as the expression of Hexon in the AdSurp-EGFP group, were confined within the cancer cells (Fig.4A-B), indicating that oncolytic adenoviruses can target the Survivin-positive HCC cells and replicate in tumor cells with specific expression of the therapeutic gene Hsp70.

The mouse blood was collected to prepare the sera. The expression of Hsp70 protein in sera was detected with an ELISA. With the selective replication of AdSurp-Hsp70 in the tumor cells, the Hsp70 level in mouse sera was increased. The Hsp70 content in mice with the strongly positive Survivin tumor xenografts was significantly higher than that in mice with the weakly positive Survivin tumor xenografts (Fig. 4C).

### Oncolytic adenovirus-mediated Hsp70 expression induced CIK cell infiltration and inhibited tumor cell proliferation

**Figure 5:**
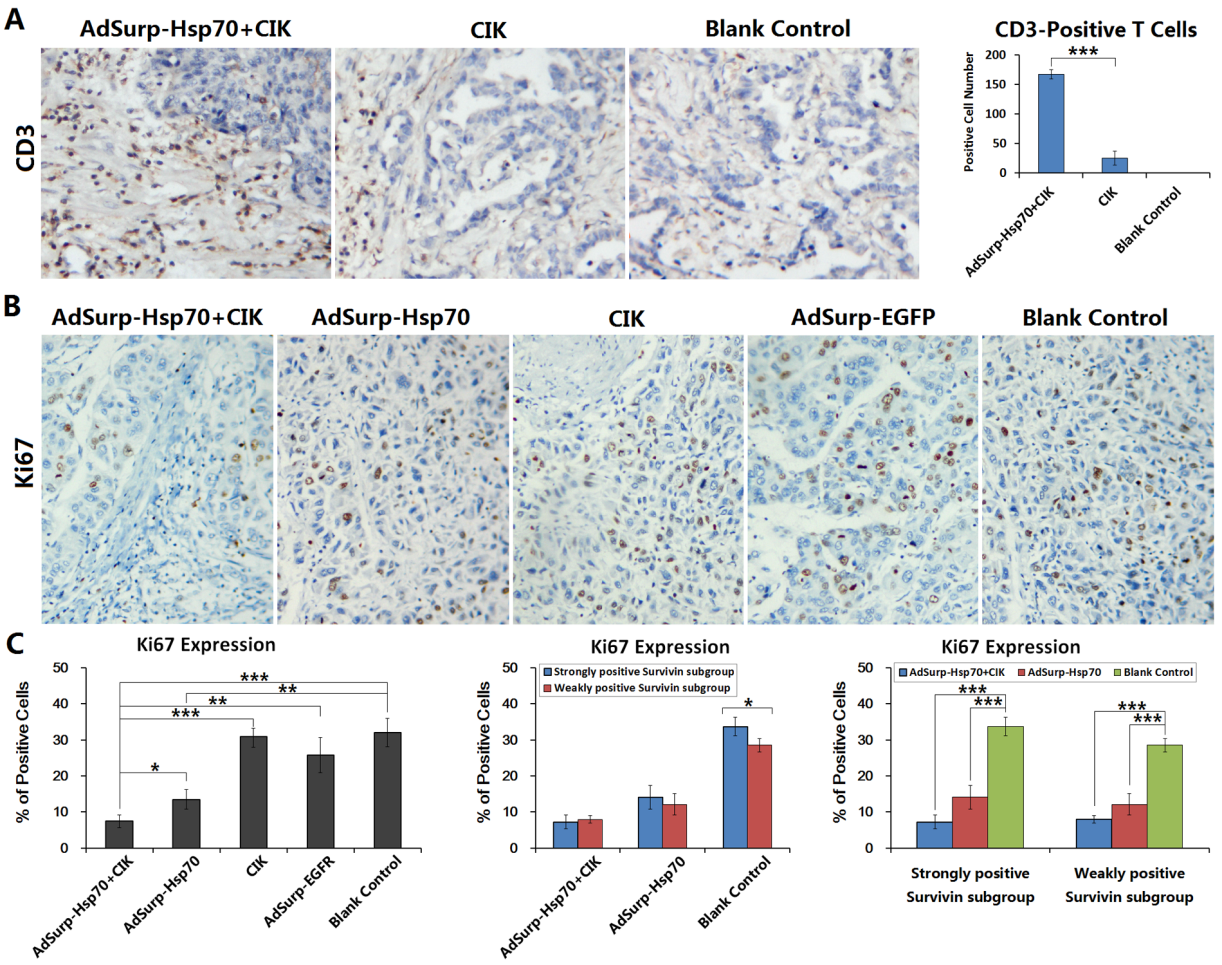
CIK cell infiltration in tumor stroma and Ki67 expression in cancer cells (A) Tumor xenografts were collected to prepare the paraffin-embedded sections as aforementioned. Immunohistochemical staining was performed to observe the number and distribution of the infiltrated CD3+ T cells in tumor stroma. For each slice, the number of positive cells was counted within 5 medium-power magnification fields of view (20× objective lens) under microscope. The results were determined by scoring the stained cell ratio and the staining intensity; original magnification: ×200; ***P<0.001. (B) Immunohistochemical staining was performed to observe the expression of the proliferating cell nuclear antigen Ki67 in tumor cells; original magnification: ×200. (C) The number of positive cells was counted within 5 medium-power magnification fields of view (20× objective lens) under microscope. Each experimental group was divided into a weakly positive Survivin subgroup and a strongly positive Survivin subgroup to further compare Ki67 expression in cancer cells; *P<0.05, **P<0.01, ***P<0.001.

Because the anti-tumor effect of Hsp70 is closely related to the regulation of immune functions, the infusion of CIK cells was conducted to partially reconstruct the cellular immune function in the tumor xenograft nude mouse models. After treatment with CIK infusion and adenovirus injections, the tumor xenograft tissues were collected to prepare the sections. The results of immunohistochemical staining showed that a large amount of CD3+ T cells had infiltrated the tumor stroma of xenografts in the AdSurp-Hsp70+CIK treatment group, these cells were distributed in and around the cancer nests, particularly in the strongly positive Survivin tumor xenografts. In the CIK group, less T cells had infiltrated the xenograft tumor stroma, and they had a scattered distribution. The blank control group showed no lymphocytic infiltration (Fig. 5A).

The proliferative activity of cancer cells was determined by immunohistochemical staining of the proliferating cell nuclear antigen Ki67. Compared with the control group, the level of positive Ki67 expression in the AdSurp-Hsp70+CIK treatment group was the lowest, indicating that the AdSurp-Hsp70+CIK treatment had the best inhibition efficiency against the proliferating activity of cancer cells. The next lowest level of positive Ki67 expression was found in the AdSurp-Hsp70 treatment group, while the CIK and AdSurp-EGFP groups showed no significant differences in the levels of Ki67 expression compared with the control group (Fig. 5B). A further analysis of the differences between the two subgroups after the treatments of AdSurp-Hsp70+CIK or AdSurp-Hsp70 found that the level of Ki67 expression in the strongly positive Survivin tumor xenografts was not significantly different from the level of Ki67 expression in the weakly positive Survivin tumor xenografts (Fig. 5C). Once compared with the corresponding subgroup in the blank control group, the level of Ki67 expression was decreased significantly both in the strongly positive Survivin tumor xenografts and weakly positive Survivin tumor xenografts after therapy.

## DISCUSSION

HCC is highly malignant and seriously affects the quality of life and the survival of patients. Currently, the comprehensive therapeutic strategy combined with biological treatment has become an important means to improve the treatment efficacy and to prolong the survival of patients with HCC. In our previous studies, the tumor-specific Survivin gene promoter was used to regulate the target replication of adenoviruses and to mediate the specific expression of anti-tumor genes for the anti-tumor experimental study. Oncolytic adenovirus vectors can specifically replicate in various tumor cells, including HCC, gastric cancer, and gallbladder carcinoma, to lyse cancer cells. They can also mediate the targeted expression of the anti-tumor genes in cancer cells, resulting in a significant anti-tumor effect without significant toxicity exposure for normal cells [1,2,4]. Because Survivin is barely expressed in normal tissues but is highly expressed in most malignant tumors, its expression is highly selective, making it a broad spectrum molecular target for the diagnosis and treatment of tumors [16]. Accordingly, the oncolytic adenoviruses regulated by the Survivin promoter can initiate the replication mechanism in the Survivin positive tumor cells and lyse the cancer cells. Meanwhile, the copy number of the anti-tumor gene is increased, leading to an efficient overexpression. This targeted treatment strategy achieves a synergy from the oncolytic effect through viral replication and the anti-cancer effect through transgene expression, and the synergistic result further enhances anti-cancer efficacy and safety. This strategy is expected to apply in the comprehensive treatment of a broad spectrum of cancers.

Development of a biotherapy strategy for tumors requires establishing an adequate animal experimental platform for preclinical screening and optimization, to reduce or avoid the risk of clinical trials in humans. In our previous studies, the tumor cell lines of HCC and gastric cancer were used to establish the xenograft models in nude mice, which were applied to verify the efficacy and safety of the gene therapy strategy with oncolytic adenoviruses. Although such models are common techniques for developing anticancer drugs and selecting treatment strategies, there are obvious shortcomings. In the processes of establishing and passing the tumor cell lines, changes in genetic characteristics and biological behaviors may occur in cells. Because a cell clone is from a single source, the heterogeneity of tumor cells in the patient is always lost in cell lines, so it cannot truly reflect the individual differences in tumors responding to a treatment. This may result in a bias of anti-tumor drug selection or treatment strategy evaluation. To construct an experimental technology platform for development of new drugs and technologies that is more similar to the clinical conditions between the preclinical study and clinical trial, the PDTX models were established. The PDTX model can not only reproduce the tumor characteristics of every patient according to their molecular biology and histopathology, but it can also retain the heterogeneity of tumors. Each animal was transplanted with a tumor from a patient, so each animal represents an individual patient, which can more accurately simulate the individual differences in the patients with primary tumors to reflect the actual sensitivity of different patients responding to the treatment. Additionally, the PDTX model can also transplant the tumor from a patient into multiple animals to implement a synchronized intervention in different groups. This superiority can overcome the problem that a patient cannot receive two or more treatments in a clinical trial, and is conductive to the selection and optimization of individualized treatments [12,13,17,18]. However, the PDTX model also has some shortcomings. It must be established using immunodeficient mice, so this model cannot be applied in the study of tumor immune function or the selection of immune gene therapy strategy. In our preliminary study, the intravenous infusion of CIK cells was performed to partially reconstruct the immune regulatory function in nude mice, which successfully overcame this defect of PDTX model and provided the conditions for studying individualized anti-tumor immune gene therapy.

To further verify the anti-tumor activity of gene therapy strategy with the expression of immunomodulatory factor Hsp70 in Survivin targeting oncolytic adenovirus, this study used the surgical specimens from 10 clinical cases of HCC to establish PDTX models in 50 nude mice with 4 parallel experimental groups and a control group for each tumor, so that the process of a clinical trial in patients could be simulated. Hsp70 has an immunomodulatory effect and plays important roles in the immune response, the host resistance to infection, and the autoimmune response [8,19]. Hsp70 can trigger the specific immunity against tumor cells. Through the activation of NK cells and γ/δ T cells, Hsp70 can induce an immune response restricted by non-major histocompatibility complexes (MHCs) and activate the complement system to promote the release of multiple cytokines, playing an anti-tumor effect [20] Through the intravenous infusion of CIK cells into mice in the PDTX models, the *in vivo* immune function was partially reconstructed in immunodeficient mice. Combined with the treatment of oncolytic adenovirus AdSurp-Hsp70, Hsp70 was specifically expressed in cancer tissue, which allowed the exogenous CIK cell chemotaxis to concentrate in cancer tissue and induced the infiltration of CD3+ T cells in tumor stroma, showing anti-tumor activities. Meanwhile, the selective replication of oncolytic adenovirus AdSurp-Hsp70 also exerted a certain degree of oncolytic effect. The results showed that either the immune response caused by the infusion of CIK cells alone or the oncolytic effect due to the replication of oncolytic adenovirus (AdSurp-EGFP) alone was very limited. When the combined intervention of AdSurp-Hsp70 injection and CIK cell infusion was conducted, the resulting tumor suppression effect was significantly enhanced, suggesting that in the immune reconstitution models, the high expression of Hsp70 can induce the host immune response to synergistically exert anti-tumor activity together with the oncolytic effect produced by viral replication. The analyses of Survivin expression in cancer tissues of the parallel experimental groups showed that the combined therapeutic intervention strategy with oncolytic adenovirus AdSurp-Hsp70 injection and CIK cell infusion had a more significant effect on tumor xenografts with high expression of Survivin. In this parallel grouping experiment, a synchronized parallel controlled experimental study was performed for each tumor case, which is achievable only in the PDTX models and the results are more accurate and reliable.

In summary, the PDTX models for HCC were applied in this study to verify the targeting and effectiveness of the therapeutic strategy of anti-tumor immune factor Hsp70 expression in the Survivin promoter-regulated oncolytic adenovirus combined with CIK infusion. Our experimental results confirmed that this therapeutic strategy can be synergistic with multiple anti-tumor mechanisms to exert effective anti-tumor activities, showing a significant inhibitory effect against the growth of HCC xenografts, especially for Survivin positive HCC. Accordingly, the established therapeutic strategy of Survivin targeting oncolytic adenovirus is feasible and effective. This is very significant for guiding clinical treatment for highly malignant HCC and other tumors with high Survivin expression.

## MATERIALS AND METHODS

### Amplification and purification of adenoviral vectors

Oncolytic adenoviruses, AdSurp-Hsp70 and AdSurp-EGFP, were amplified in HEK293 cells. The adenovirus DNA was extracted using a QIAamp DNA Blood Mini Kit (QIAGEN Inc., Shanghai, China) according to the kit’s instructions. The specific primers for E1 region (upstream: 5′-GTG TAT TTA TAC CCG GTG AG-3′, downstream: 5′-TGG AAG ATT ATC AGC CAG TAC-3′) and Hsp70 (upstream: 5′-CCC AAG CTT ATG GCC AAA GCC GCG GCG-3′, downstream: 5′-GCG TCG ACC TAA TCT ACC TCC TCA ATG GTG GG-3′) were used to identify the virus. After the identification, the viruses were purified using conventional cesium chloride gradient centrifugation, and the viral titer was determined using the 50% tissue culture-infective dose (TCID50) method.

### Preparation of mouse CIK cells

The CIK cells were derived from normal BALB/C mice. The methods of cell harvest, culture and *in vitro* activation were described in detail in the literature [21].

### Identification of Survivin expression in HCC specimens

HCC specimens and paracancerous liver tissues were collected from 10 patients undergoing clinical operation in March 2014. All patients, male, average age 49 years old (42-57), were confirmed to be positive for HBsAg in sera and did not treated with chemotherapy or radiotherapy before surgery. The resected lesions were diagnosed pathologically as HCC, and the tumor stage was classified according to the Barcelona Clinic Liver Cancer System (BCLC), in which there were 2 in Stage A, 5 in Stage B and 3 in Stage C. A sample of 1 cm^3^ fresh tumor tissue without necrosis was quickly excised from every patient for transplantation to the animal model. Another specimen of tumor tissues was fixed in 10% formalin for 6 h to prepare the paraffin-embedded sections, and the expression of Survivin was detected by immunohistochemistry with the mouse anti-human Survivin antibody at a working concentration of 1:100 and the streptavidin-peroxidase (SP) immunohistochemical kit (Fuzhou Maixin Biotechnology Development Co., Fuzhou, China). For each slice, the number of positive cells was counted within 5 medium-power magnification fields of view (20× objective lens) under microscope. The results were determined by scoring the stained cell ratio and the staining intensity. The staining intensity scores were defined as follows: staining identical to the negative control was defined as 0, while yellow, brown, and tan staining were defined as 1, 2, and 3, respectively. The staining scores of positive stained cell ratio were defined as follows: the score was 0 when cells were all negatively stained, the scores were 1, 2, and 3, respectively, if the proportion of the positively stained cells was 1/3 or less, 1/3 to 2/3, and 2/3 or more. The sum of above 2 scores was grouped into 3 categories: 0, 1-3, and 4-6 corresponded to negative, weakly positive, and strongly positive, respectively.

### Establishment of xenograft models in nude mice and viral treatment

Eighty healthy purebred BALB/C nude mice, male, 4 weeks old, were purchased from the Shanghai SLAC Laboratory Animal Center of Chinese Academy of Sciences (Shanghai, China). The fresh HCC tissues were placed in DMEM (Dulbecco’s Modified Eagle Medium) containing 10% fetal bovine serum (FBS), and quickly cut to a depth of 2 mm in each piece using a sharp blade, which was subcutaneously buried in the right axilla of the nude mice using a trocar puncture. The tumor tissue from each patient was transplanted to 8 nude mice, in which 3 mice were prepared to replace the mice that failed to form xenografts, and the other 5 mice were assigned to 5 experimental groups (AdSurp-Hsp70+CIK, AdSurp-Hsp70, CIK, AdSurp-EGFP, and blank control group), that is, the 10 tumor xenografts of the 10 nude mice in each experimental group represented the 10 patients. The mice were continuously fed, and the growth of the tumors was regularly observed.

Three weeks after the transplantation, the tumor xenografts had grown in size to a diameter of approximately 6-10 mm. For the treatment, the mice in the AdSurp-Hsp70+CIK and CIK groups were infused with the pre-cultured CIK cells through the tail vein to a concentration of 10^7^ cells for each mouse. Subsequently, the corresponding viral treatment was given based on the grouping. A total of 2×10^8^ pfu/50 μL of the virus was injected directly into the tumor at multiple sites every other day for a total of 5 times. The blank control group was injected with the viral preservation solution (10 mmol/L Tris-HCl pH 8.0, 2 mmol/L MgCl_2_, 4% sucrose) instead of the viral injection at a volume of 50 μL for a total of 5 times. The tumor size was measured regularly, and the tumor volume was calculated with the formula of “a×b^2^×0.5” (a: maximum diameter, b: minimum diameter), resulting in the tumor growth curve.

### Detection of viral replication activity and gene expression

In the *in intro* experiments, the normal liver cell line L02, HCC cell lines SMMC-7721 and BEL-7404 (Shanghai Institute for Biological Science, Chinese Academy of Science, Shanghai, China) were seeded into 24-well plates at a concentration of 1×10^5^ cells/well and infected with adenoviruses AdSurp-EGFP and AdSurp-Hsp70, respectively, at a multiplicity of infection (MOI) of 1 pfu/cell and cultured for 48 h. The AdSurp-EGFP-infected cells were counted the percentages of EGFP-positive cells under a fluorescent microscope. The AdSurp-Hsp70-infected cells were harvested and extracted total protein, which were then subjected to Western blotting to determine Hsp70 expression.

In the animal experiments, after the observation period, the mice were anesthetized with an intraperitoneal injection of 3% sodium pentobarbital. The tumor xenografts were collected, weighed, and fixed in 10% neutral buffered formalin for 6 h to prepare the paraffin-embedded sections. Immunohistochemical staining was performed to locate the expression of adenoviral capsid protein Hexon with the mouse anti-Hexon monoclonal antibody at a working concentration of 1:200), Hsp70 with the mouse anti-Hsp70 monoclonal antibody at a working concentration of 1:100 (Santa Cruz Biotechnology, Inc., Santa Cruz, CA, USA) and Survivin with the mouse anti-human Survivin antibody at a working concentration of 1:100. The positive judgment standard for each slice was stated as aforementioned. The blood was collected from mice, and the expression of Hsp70 in serum was detected by an enzyme-linked immunosorbent assay (ELISA), according to the instructions of Hsp70 ELISA kit (Stressgen Biotechnologies Corp., Victoria, Canada).

### Identification of CIK infiltration and the cancer cell proliferation

The number and distribution of CD3+ infiltrated T cells in the tumor stroma were determined by immunohistochemistry on xenograft sections with the rabbit anti-mouse CD3 antibody (Santa Cruz Biotechnology, Inc.; at a working concentration of 1:200). The proliferation activity of cancer cells was immunohistochemically determined by the expression of proliferating cell nuclear antigen Ki67 with the rabbit anti-Ki67 polyclonal antibody (Abcam, Cambridge, MA, USA; at a working concentration of 1:200). The positive judgment standard for each slice was stated as aforementioned.

### Statistical analysis

All experimental data were presented as the mean ± standard deviation (*x̅*±s). Comparisons of the paired data were carried out using *t*-test and chi-square test, and comparisons among multiple groups were carried out using an ANOVA. All data were analyzed with the SPSS 13.0 software package, and *P*<0.05 indicates a statistically significant difference.
